# Clinical efficacy of short-course chemotherapy combined with topical injection therapy in treatment of superficial lymph node tuberculosis

**DOI:** 10.18632/oncotarget.22492

**Published:** 2017-11-18

**Authors:** Huiru An, Zhongyuan Wang, Hongbing Chen, Tao Wang, Xinjing Wang, Lin Liu, Xiao Liu, Jing Xu, Luxing He, Kai Zhang, Hongyan Zhang, Xinying Liu

**Affiliations:** ^1^ Military Institute of Tuberculosis, Beijing, 100091, China

**Keywords:** tuberculosis, lymph node, short-course chemotherapy, regional injection

## Abstract

To evaluate the clinical efficacy and safety of short-course chemotherapy combined with regional injection therapy in the treatment of superficial lymph node tuberculosis. 201 patients diagnosed with superficial lymph node tuberculosis were retrospectively analyzed. All patients were randomly divided into the study (*n* = 100) and control groups (*n* = 101). In the study group, the patients received 6-month chemotherapy with isoniazid (H), rifampin (R) and ethambutol (E) (6HRE) in combination with regional injection of streptomycin, and their counterparts in the control group underwent systemic regime of 3HRZE/6HRE. In the study group, the overall cure rate was calculated as 98% and the recurrence rate was 2%. Twenty-four of 25 nodular type patients and 36 among 37 inflammatory type patients were recovered and discharged. One patient with huge nodular type mass was treated for 4 months and the mass size was slightly reduced. In the control group, the overall cure rate was 48.5% and the recurrence rate was 7.9%. The recurrent patients were further administered with regional injection of streptomycin based upon the chemotherapy regime until they were recovered. Combined therapy of systemic chemotherapy and regional injection of streptomycin is probably an efficacious and safe approach in the treatment of superficial lymph node tuberculosis, which remains to be validated by more investigations.

## INTRODUCTION

Superficial lymph node tuberculosis is the most common extra-pulmonary manifestation of tuberculosis [1]. In a series of randomized clinical trials, the British Thoracic Society (BTS) demonstrated that the duration of treatment for this disease could be shortened from 18 months to 9 months initially and later to 6 months [2]. Differential diagnosis includes other infections, neoplasia, congenital conditions in the head and neck and rarely, drug reactions. Diagnosis should be made on the basis of histological evidence after lymph node biopsy. Diagnosis made on clinical grounds has poor specificity and will result in a great degree of over diagnosis.

Systemic chemotherapy and surgical resection of the lymph node lesions yield relatively limited clinical efficacy, whereas relatively high recurrence rate [3]. In view of the increasing incidence of drug resistance, bacterial confirmation and drug susceptibility test should be performed whenever possible.

Even after the delivery of effective chemotherapy, it still poses considerable problems to the clinical diagnosis and management of this disease [4]. Patients constantly present with a painless lymphadenopathy of the superficial lymph nodes of insidious onset, which may evolve into abscess and sinus formation if left untreated or even treated regularly.

To enhance the clinical efficacy and reduce the recurrence rate, 201 patients diagnosed with superficial lymph node tuberculosis were treated with conventional therapy and systemic short-course chemotherapy combined with regional injection of streptomycin in this clinical trial, aiming to statistically compare the clinical efficacy and safety between these two approaches.

## RESULTS

In the study group, 98 patients were effectively treated with a cure rate up to 98%. The frequency of injection of streptomycin was ranged from 2 to 40 times, 12 times on average for each patient. One uremia patient presented with tinnitus after hemodialysis. No other toxic reactions were observed in the remaining cases following the local injection of streptomycin. For the abscess type patients, the abscess pus was completely removed and regional injection of streptomycin was delivered. They were completely healed. Twenty-four of 25 nodular type patients and 36 among 37 inflammatory type patients were recovered and discharged. One patient with huge nodular type mass was treated for 4 months and the mass size was slightly reduced. Subsequently, the therapy was terminated. One inflammatory type patient received hemodialysis and the mass size was slightly decreased. However, the therapy was discontinued after the presence of tinnitus and lip numbness. During 2-year follow-up, merely 2 patient had recurrence of superficial lymph node tuberculosis in other site. The patient was successfully healed after regional administration of streptomycin.

In the control group, only 49 of 101 patients were healed after receiving systemic chemotherapy. The cure rate of nodular type patients was the highest up to 92.3% (24/25), which was similar to the cure rate in the study group. Nevertheless, the cure rate of the abscess and inflammatory type patients in the control group was significantly lower compared with that in the study group. For 10 ulcer type patients, the sinus tract was healed and the symptoms were alleviated after systemic chemotherapy and dressing change in 5 cases (50%). During the follow-up, 8 patients had recurrence of superficial lymph node tuberculosis. Fifty-two patients with enlarged mass size and deterioration were subsequently topically injected with streptomycin based upon the systemic chemotherapy regimen. Subsequently, these patients were successfully healed and discharged, as illustrated in Tables 1 and 2.

**Table 1:** Comparison of clinical efficacy between two groups

Group	Efficacious (*n*)/Total (*n*)	Inefficacious (*n*)/Total (*n*)
Study group (*n* = 100)	98/100 (98.0%)^*^	2/100 (2%)^*^
Control group (*n* = 101)	49/101 (48.5%)	52/101 (51.5%)
χ^2^		23.52
*P*		0.012

**Table 2:** Comparison of clinical efficacy in patients with different types of superficial lymph node tuberculosis between two groups

Type	Group	Efficacious (*n*)/Total (*n*)	Inefficacious (*n*)/Total (*n*)
Nodular type	Study group	24/25 (96%)	1/25 (4%)
	Control group	24/26 (92.3%)	2/26 (7.7%)
χ^2^			21.04
*P*			0.511
Inflammatory type	Study group	36/37 (97.3%)	1/37 (2.7%)
	Control group	14/36 (38.9%)	22/36 (61.1%)
χ^2^			25.11
*P*			0.025
Abscess type	Study group	30/30 (100%)^*^	0/30 (0%)^*^
	Control group	6/29 (20.7%)	23/29 (79.3%)
χ^2^			29.36
*P*			0.014
Ulcer type	Study group	8/8 (100%)^*^	0/8 (0%)^*^
	Control group	5/10 (50%)	5/10 (50%)
χ^2^			32.05
*P*			0.012

The clinical efficacy between two regimens in the treatment of nodular type patients did not significantly differ by using the Fisher exact probability test (*P* > 0.05). Statistical significance was noted between the study and control groups in terms of the management of inflammatory type patients by using the chi-square test (*P* < 0.05). These statistical data revealed that the clinical efficacy between two regimens significantly differed in the treatment of abscess type patients (χ^2^ = 23.52, *P* < 0.05).

## DISCUSSION

Tuberculous lymphadenitis is the most common extrapulmonary manifestation of tuberculosis [5]. Short course chemotherapy is now well established in treating pulmonary tuberculosis, but there is little information about its value in lymph node disease in children. In a controlled clinical trial in Britain the British Thoracic Society found that a 9-month regimen of rifampicin and isoniazid supplemented by ethambutol for the first two months was as effective as a conventional 18 month regimen in adults with tuberculous lymphadenitis [2]. Previous studies have demonstrated the success of a 6 month regimen. Previous reports on the management of this disorder had dealt with conventional regimens of longer duration with or without surgery. All these studies were conducted in adults [6–9].

Nevertheless, no unified standards have been established in the timing and mode of surgery. In addition, a majority of patients constantly suffer from varying postoperative complications, such as incision disunion, fistula formation, sinus tract and scarring, *etc*. More importantly, the high recurrence rate severely affects the quality of life of patients.

At present, several scholars have attempted to treat the tuberculosis in the pattern of traditional Chinese medicine [10]. However, the clinical efficacy, long-term safety and the exact pathogenesis remain to be elucidated. Thus, larger sample size and profound investigations are urgently required to evaluate the clinical efficacy and safety of traditional Chinese medicine therapy.

Currently, chemotherapy is still the major treatment of tuberculosis with higher efficacy and less postoperative complications compared with alternative regimens. However, the course of chemotherapy is relatively long and even the recurrence rate is relatively high [11]. These issues are challenges to the physicians. In this study, the clinical efficacy of the systemic chemotherapy along was also investigated and the results demonstrated the clinical efficacy was significantly lower in terms of the management of superficial lymph node tuberculosis. Since the lymph node masses are surrounded by integrated envelope. Single application of systemic anti-tuberculosis medication is difficult to accumulate the sufficient drug concentration to eliminate the bacteria and thereby it fails to thoroughly and effectively lower the regional concentration of pathogenic bacteria. To enhance the drug concentration in the lymph node tissues, regional medicine administration should be delivered. Moreover, the lymph node lesions should be eliminated in a timely fashion. All these interventions should be implemented to enhance the clinical efficacy and reduce the recurrence rate.

In this clinical trial, patients diagnosed with superficial lymph node tuberculosis received short course 6HRE-based chemotherapy in combination with regional injection of streptomycin, which significantly shortens the course of treatment. The other advantage of this combined therapy is to considerably reduce the recurrence rate. Topical drug administration could penetrate towards the surrounding lymph nodes and eventually reach the distant lymph nodes to thoroughly eliminate the bacteria. For a minority of patients with mediastinal lymph node tuberculosis, the mass size was significantly reduced after topical drug administration. In this study, chemotherapy combined with regional drug injection yielded high clinical efficacy. Subsequently, these therapeutic regimens were strengthened to maintain the clinical efficacy. Three different medicine were administered after the abscess pus was completely eliminated, which contributed to the rapid delivery of drugs to the affected lymph node and enhanced the clinical efficacy. Streptomycin has been utilized as a conventional anti-tuberculosis drug [12–14]. It yields high efficacy in eliminating extracellular tubercidin bacillus and slight adverse events. Streptomycin has been widely applied in the treatment of tuberculosis by clinicians. However, it can be administered merely via intramuscular route. In addition, it is likely to cause solid masses surrounding the injection site, thereby affecting the chemotherapy compliance, medication absorption rate and blood drug level. Besides, the clinical application of this drug is limited due to the adverse reaction and high drug resistance [15]. To reduce the risk of adverse events, regional injection rather than systemic administration of streptomycin was chosen. The total drug quantity was significantly decreased, which considerably reduced the incidence of adverse reactions and significantly elevated the compliance of chemotherapy. In addition, streptomycin is highly efficacious for eliminating the extracellular tubercle bacillus and maintained the high concentration of streptomycin within the lymph node tissues, which can rapidly and thoroughly eliminate the tubercle bacillus within the lymph nodes. Compared with the isoniazid, rifamycin and amikacin, streptomycin has higher clinical efficacy [16]. The regional concentration of streptomycin is significantly higher compared with that of other drugs. The high drug concentration is the advantage of streptomycin, which can significantly enhance the clinical efficacy [17]. Even if drug resistance occurs during the treatment, it merely targets the streptomycin and does not affect the clinical efficacy of other drugs. Therefore, the application of streptomycin is more efficacious compared with that of the use of isoniazid and other drugs. Taken together, regional injection of streptomycin combined with 6HRE systemic chemotherapy is an efficacious and safe approach in the treatment of superficial lymph node tuberculosis. In addition, it significantly shortens the course of treatment, averts the use of surgery, strengthens the patients’ compliance towards the chemotherapy and significantly decreases the recurrence rate. There are several limitations to be acknowledged. The kidney injury, diabetes mellitus with cellutitis and pain control should be further analyzed. The long-term clinical efficacy and safety of this combined regimen remain to be validated by more investigations.

## MATERIALS AND METHODS

### Baseline data

Among 201 patients diagnosed with superficial lymph node tuberculosis, 72 cases were hospitalized and 129 were outpatient cases, 41 male (20.4%) and 160 female (79.6%). All patients were aged 13–82 years with a mean age of 32.2 years. Three patients presented with armpit focus, one with inguinal lymph node and the remaining cases had cervical lymph node tuberculosis. The tumor size was >2 cm for all cases. The maximal tumor size was measured as 13 cm. All patients were negative for skin test of streptomycin administration. All 201 patients were randomly assigned into the study (*n* = 100) and control groups (*n* = 101). The study procedures were approved by the ethics committee of our hospital. Diagnostic criteria: Among 201 cases, 47 were positive for acid-fast staining of the lymph node puncture fluid. Aspiration biopsy of the lymph node detected the tuberculous granuloma and dry cheese-like necrosis in 154 cases. All patients were positive for PPD skin test.

### Classification of superficial lymph node tuberculosis

According to the pathological findings and clinical manifestations, superficial lymph node tuberculosis was classified into four types [18]. Nodular type was pathologically manifested as tuberculous granuloma, no swelling, fever or pain. The masses had clear margin in an isolated or bead shape. Fifty-one patients were diagnosed with nodular type with 25 in the study group and 26 in the control group. Inflammatory type was pathologically characterized with caseous necrosis or acute and chronic inflammatory cell infiltration. The mass surface was accompanied with swelling, pain and other inflammation manifestations. The tumor texture was moderate, unclear margin and significant adhesion. Seventy-one cases had inflammatory type with 37 in the study group and 36 in the control group. Abscess type was pathologically manifested as a large quantity of pus and white blood cells with fluctuation sense. The pus was removed in 59 patients including 30 in the study group and 29 in the control group. Ulcer type was characterized with epidermis ulceration, sinus tract formation, which constantly progressed from the inflammatory type or abscess type. eighteen patients presented with ulcer type including 8 in the study group and 10 in the control group.

### Therapeutic procedures

In the study group, systemic chemotherapy consisting of isoniazid, rifampicin and ethambutol was adopted, and intra-lymph node injection of streptomycin was administered after the removal of pus. Each cycle of injection of streptomycin was delivered at a dosage of 1 g with 2 ml of sterile water. For the mass size <3 cm, the injection of streptomycin was administered once weekly, and twice weekly for those with tumor size >3 cm. For those patients with mass size >6 cm or significant abscess, the abscess pus was removed and injection of streptomycin was given for 2 to 20 times, 12 times on average, even once daily until the mass size was reduced obviously. For those with mass diameter >6 cm, the cotton soaked with streptomycin (a dose of 10 mg/kg) was placed into the masses and the necrotic masses were removed. In the control group, systemic regimen was applied as 3HRZE/6HRE.

### Evaluation of clinical efficacy

The clinical efficacy was assessed according to the findings of ultrasonic examination. Efficacious was defined as the reduction of lymph node mass exceeded 1/2 and over 2/3 of the ulcer surface was healed. Inefficacious was defined as the reduction of lymph node mass <1/2 and less than 2/3 of the ulcer surface was cured.

### Statistical analysis

All data were analyzed by using SPSS 19.0 statistical software (SPSS Inc., Chicago, IL, USA). Comparison among two groups was performed by chi-square test. A *P* value of less than 0.05 was considered as statistical significance.

## Abbreviations

6HRE: 6-month chemotherapy with isoniazid (H), rifampin (R) and ethambutol (E)
BTS: the British Thoracic Society
